# Domestic violence against women in Shiraz, South-western Iran

**DOI:** 10.5249/jivr.v11i2.1238

**Published:** 2019-07

**Authors:** Bahareh Moazen, Alireza Salehi, Maryam Soroush, Hossein Molavi Vardanjani, Amir Zarrinhaghighi

**Affiliations:** ^*a*^MPH Department, Shiraz Medical School, Shiraz University of Medical Sciences, Shiraz, Iran.; ^*b*^Research Center for Traditional Medicine and History of Medicine, Shiraz University of Medical Sciences, Shiraz, Iran.; ^*c*^Academic Center for Education, Culture, and Research (ACECR)-Fars Branch, Iran.; ^*d*^Student Research Committee, Shiraz University of Medical Sciences, Shiraz, Iran.

**Keywords:** Domestic violence, Public health, Women’s health

## Abstract

**Background:**

Domestic violence against women can lead to short and long term health-related issues. We aimed to estimate the prevalence of domestic violence against married women and its correlates in southwestern, Iran.

**Methods:**

A population-based survey was carried out from February 1st to May 30th, 2018 in Shiraz, Iran. Currently married or recently separated/divorced women who visited healthcare centers were voluntarily interviewed. World Health Organization (WHO) standard domestic violence questionnaire was used to measure domestic violence. Hence, its prevalence and correlates were assessed. Data were analyzed using multivariable logistic regression.

**Results:**

Lifetime prevalence of overall, mental, physical, and sexual domestic violence were 54.5% (95% CI: 49.6, 59.4), 52.0% (95% CI: 47.1, 57.0), 18.2 % (95% CI: 14.4, 22.0) and 14.0 % (95% CI: 10.6, 17.4), respectively. Living separately, increasing spouse’s age, the higher number of children, rental housing, middle to low monthly income, and history of domestic violence in the family of husband and/or wife had a positive correlation with domestic violence in some categories.

**Conclusions:**

More than half of the married women in southwestern Iran are experiencing domestic violence, and mental domestic violence is the most common type. Economic instability and witnessing domestic violence in childhood are the most correlates of domestic violence. Family violence preventive services and other population-based measures are highly necessary for this region.

## Introduction

**Domestic Violence**

Domestic violence (DV) has been defined as the violent and dominant behaviour of a family member against other members of the same family. Usually, women and girls are the first victims of DV.^1^ Violence against women is any act of gender-based violence that leads to a woman's physical, sexual or psychological harm, resulting in her suffering or forced deprivation of her individual or social freedom.^2^ The psychological injuries associated with violence can be feelings, such as helplessness, lack of confidence, anxiety, depression, and suicide. Also, physical disabilities, chronic headaches, drug use, and mental disorders are expected^3,4^

Domestic violence is mainly categorized into three groups: Mental, Physical and Sexual Violence.^5^

**Literature Review**

In a large population study by the World Health Organization (WHO) in 15 regions of 10 countries 2000-2003, on 24096 women, DV rate was 15-71%. According to this study, physical and sexual violence in the world is widespread.^5^

In a meta-analysis study conducted in Iran, April 2017 on 31 articles on DV against women (from 2000 to 2014), it was estimated that DV prevalence in Iran is 66%. Prevalence rate in the Eastern region of Iran was 70%, West 75%, North 62%, South 70%, and in the Central region 59%, respectively.^6^

In a study by Kargar Jahromi et al. in Jahrom city (Fars province), the prevalence of physical, sexual and emotional violence against women was 16.4%, 18.6% and 44.4%, respectively. In this study, domestic violence against women was positively associated with factors such as younger women and men, low duration of marital years, and low level of education amongst men and women.^7^

In the study by Shayan et al. in Shiraz, on 197 women who had referred to Shiraz Forensic medicine, more than 50% were subjected to DV, and had general health problems. They were also suffering from depression and anxiety.^8^

**Aim of the study**

Considering the significant effects of DV on familial and social health, as well as lack of sufficient evidence in Fars province, especially Shiraz, situation analysis might facilitate evidence-based health policy making. Considering higher prevalence of DV among families with lower economic condition in several previous studies as well as the current economic instability due to economic sanctions in Iran,^9,10^ this study can provide an update on the prevalence of DV.. This study was conducted to determine the prevalence of domestic violence, and its correlates against married women who visited health centers in Shiraz, 2018. 

## Materials and Methods

**Setting**

The study was a population-based survey, carried out from February 1st to May 30th, 2018, in Shiraz., the capital city of Fars province, located in the southwestern part of Iran (29.5929° N, 52.5836° E) with a population of 1869000, according to the Population and Housing Census report from the Statistical Center of Iran in 2016. Based on this census, 50.43% of Shiraz’s inhabitants are men and 49.57% women. Also, among people over the age of 6, 94.83% of men and 91.43% of women are literate. Furthermore, among the population over the age of 10, 60.53% of men are married, 1.46% divorced, 0.78% widowed, and 37.21% are single. Also, 60.89% of women are married, 2.68% divorced, 7.24% widowed, and 29.18% bachelorette.^11^

**Participants**

Currently married or recently separated/divorced women who visited health centers for a routine check-up, neonatal, child or maternal healthcare or as patient companion voluntarily participated in this study. Bachelorettes were excluded from the survey. All the women who entered the study gave their informed consent verbally. The study was approved by the local Ethics Committee of Shiraz University of Medical Sciences (Approval Code: IR.SUMS.MED.REC.1398.77).

**Sampling**

In this study the sample size was estimated at least 430 participants using the Cochran sample size formula by assuming 6% precision, 5% type 1 error, 75% prevalence and a response rate of 80%.

In this study, a multistage cluster sampling was done. All 10 Shiraz municipality districts (only urban) are listed as sampling stratum. Public and private sectors were considered as sampling districts to provide a representative sample of people with high or low socioeconomic status. From each district, 1 public and 1 private clinic was randomly selected, of which clinic 20 women were interviewed.

**Variables**

DV and marital status, age, spouse’s age, years of marriage, number of children, housing (own or not own), work status (housewife or employed), spouse working status (full-time, part-time and unemployed), level of education (diploma or lower, associate or bachelor, master or higher), spouse’s level of education (diploma or lower, associate or bachelor, master or higher) monthly income, age difference between spouse and wife, history of DV in the women and/or spouse’s parental family (often, sometimes, never) were measured during the interview.

**Data collection**

The adopted and standardized Persian version of the WHO standard DV questionnaire was used.^12,13^ The questionnaire includes 15 demographic questions, 11 mental DV questions, 6 physical DV questions, and 3 sexual DV questions (Appendix 1) (7 questions in terms of controlling behavior were considered as mental violence).

**Appendix 1:** Questionnaire used in the study.

Number	Type of question	Question
Question 1	Mental	Restricts contact with family
Question 2	Mental	Insists on knowing where she was
Question 3	Mental	Ignores or treats indifferently
Question 4	Mental	Gets angry when spoken with other men
Question 5	Mental	Is often suspicious of wife’s faithfulness
Question 6	Mental	Expects permission for seeking health care
Question 7	Mental	Keeps away from seeing friends
Question 8	Mental	Insults or makes her feel bad about herself
Question 9	Mental	Belittles or humiliates in front of others
Question 10	Mental	Scares or intimidates on purpose
Question 11	Mental	Threatens to hurt wife or her beloveds
Question 12	Physical	Slaps or throws something at her
Question 13	Physical	Pushes or shoves
Question 14	Physical	Hit with a fist or something else
Question 15	Physical	Kicks, drags or beaten up
Question 16	Physical	Choked or burnt on purpose
Question 17	Physical	Threaten or used a weapon
Question 18	Sexual	Physically forced to have sexual intercourse
Question 19	Sexual	Ever had unwanted sexual intercourse from fear of partner’s actions
Question 20	Sexual	Forced to do humiliating sexual activities

1. Questions asked from participants which included 11 mental, 6 physical and 3 sexual items.

The questionnaire probes the participants’ DV experience during their life as well as the last 12 months. Also, in the case of physical DV, the severity of violence is assessed by the type of violence (moderate or severe). Being slapped, pushed, shoved or something thrown at them, which is defined as moderate violence, and actions like being punched or other things, kicked, dragged, beaten up, choked or burnt on purpose, threatened with a weapon or the actual use of a weapon against them where categorized as severe physical violence.

Data were collected by two trained female healthcare nurses. Participants were oriented on how to answer the questions and then the questionnaires were filled out in a private room via face-to-face interviews. Each form was completed in approximately 10 minutes.

**Statistical Analysis**

Data were prepared using methods presented by Molavi et al.^14^ The mean and standard deviations (SD) were used for quantitative variables and relative frequency for qualitative variables. Chi-square test was used for bivariate analysis. Variable selection for multivariate analysis was done based on a conceptual framework, and P value lower than 0.25. Binary logistic regression was applied for multivariable analysis by backward elimination approach. Adjusted odds ratio (OR) and its 95% confidence interval (CI) were estimated. P values of less than 0.05 were considered to be statistically significant. All statistical analysis was done using SPSS software version 14.

## Results

In this study, response rate was 93.0%. Wives’ mean age ± SD was 38.29 ± 11.18 years, and spouses’ mean age was 42.69 ± 11.83 years. Mean marital life ± SD was calculated 14.01 ± 11.18 years. Majority of both women and men educational level was diploma with 134 (33.5%) and 111 (27.8%), respectively. Also, 264 women (66.0%) were housewives.

The lifetime prevalence of overall, mental, physical, and sexual DV were estimated at 218 (54.5%, 95%CI: 49.6, 59.4), 208 (52.0%, 95%CI: 47.1, 57.0), 73 (18.2%, 95%CI: 14.4, 22.0), and 56 (14.0%, 95%CI: 10.6, 17.4), respectively. Lifetime experience (at least one time) of moderate and severe physical DV was reported by 68 (17.0%, 95%CI: 13.3, 20.7) and 37 (9.2%, 95%CI: 6.3, 12.1) participants (Table 1). 

**Table 1:** Frequency and analysis results of qualitative parameters.

Variables		n	Experienced DV in total		Mental violence		Physical violence		Sexual violence	
			n (%)	%95CI	n (%)	%95CI	n (%)	%95CI	n (%)	%95CI
Overall		400	218 (54.5)	49.5 – 59.5	208 (52.0)	47.0 – 57.0	73 (18.2)	14.6 – 22.4	56 (14.0)	10.8 – 17.8
Marital status	Together	369	192** (52.0)	46.8 – 57.2	182** (49.3)	44.1 – 54.6	59** (16.0)	12.4 – 20.1	50 (13.6)	10.2 – 17.4
	Separated	31	26 (83.9)	66.3 – 94.5	26 (83.9)	66.3 – 94.5	14 (45.2)	27.3 – 64.0	6 (19.4)	7.6 – 37.5
Wife’s age	<30	108	52 (48.1)	38.4 – 58.0	51 (47.2)	37.6 – 57.0	11** (10.2)	5.2 – 17.5	9* (8.3)	3.9 – 15.2
	30 – 49	227	127 (55.9)	49.2 – 62.5	119 (52.4)	45.7 – 59.0	43 (18.9)	14.1 – 24.7	40 (17.6)	12.9 – 23.2
	> 50	65	39 (60.0)	47.1 – 72.0	38 (58.5)	45.6 – 70.6	19 (29.2)	18.6 – 41.8	7 (10.8)	4.4 – 20.9
Spouse’s age	< 30	46	18* (39.1)	25.1 – 54.6	17* (37.0)	23.2 – 52.4	4* (8.7)	2.4 – 20.8	6 (13.0)	4.9 – 26.3
	30 – 49	252	141 (56.0)	50.0 – 62.1	134 (53.2)	46.8 – 59.5	46 (18.3)	13.7 – 23.6	37 (14.7)	10.6 – 19.7
	> 50	99	57 (57.6)	47.2 – 67.5	55 (55.6)	45.2 – 65.5	23 (23.2)	15.3 – 32.8	13 (13.1)	7.2 – 21.4
Marital years	<10	200	104 (52.0)	44.8 – 59.1	101 (50.5)	43.4 – 57.6	29* (14.5)	9.9 – 20.2	26 (13.0)	8.7 – 18.4
	10–19	95	56 (58.9)	48.4 – 68.9	50 (52.6)	42.1 – 63.0	20 (21.1)	13.4 – 30.6	18 (18.9)	11.6 – 28.3
	> 20	105	58 (55.2)	45.2 – 65.0	57 (54.3)	44.3 – 64.0	24 (22.9)	15.2 – 32.1	12 (11.4)	6.0 – 19.1
Number of children	0	87	37* (42.5)	32.0 – 53.6	37* (42.5)	32.0 – 53.6	9** (10.3)	4.8 – 18.7	8* (9.2)	4.1 – 17.3
	1 child	117	67 (57.3)	47.8 – 66.4	63 (53.8)	44.4 – 63.1	20 (17.1)	10.8 – 25.2	15 (12.8)	7.4 – 20.3
	2 children	126	70 (55.6)	46.4 – 64.4	68 (54.0)	44.9 – 62.9	24 (19.0)	12.6 – 27.0	17 (13.5)	8.1 – 20.7
	> 3	70	44 (62.9)	50.5 – 74.1	40 (57.1)	44.7 – 68.9	20 (28.6)	18.4 – 40.6	16 (22.9)	13.7 – 34.4
Wife’s education	Diploma and lower	202	112 (55.4)	48.3 – 62.4	103 (51.0)	43.9 – 58.1	38* (18.8)	13.7 – 24.9	29 (14.4)	9.8 – 20.0
	Associate/ Bachelors	140	79 (56.4)	47.8 – 64.8	78 (55.7)	47.1 – 64.1	29 (20.7)	14.3 – 28.4	21 (15.0)	9.5 – 22.0
	Masters or higher	58	27 (46.6)	33.3 – 60.1	27 (46.6)	33.3 – 60.1	6 (10.3)	3.9 – 21.2	6 (10.3)	3.9 – 21.2
House ownership	Owning	216	100**(46.3)	39.5 – 53.2	94** (43.5)	36.8 – 50.4	38 (17.6)	12.8 – 23.3	22** (10.2)	6.5 – 15.0
	Not owning	184	118 (64.1)	56.7 – 71.1	114 (62.0)	54.5 – 69.0	35 (19.0)	13.6 – 25.4	34 (18.5)	13.1 – 24.9
Spouse’s education	Diploma and lower	194	110 (56.7)	49.4 – 63.8	104 (53.6)	46.3 – 60.8	36 (18.6)	13.3 – 24.8	28 (14.4)	9.8 – 20.2
	Associates/ Bachelors	133	74 (55.6)	46.8 – 64.2	70 (52.6)	43.8 – 61.3	27 (20.3)	13.8 – 28.1	21 (15.8)	10.0- 23.1
	Masters or higher	72	34 (47.2)	35.3 – 59.3	34 (47.2)	35.3 – 59.3	10 (13.9)	6.9- 24.1	7 (9.7)	4.0 – 19.0
Spouse’s occupation	Full–time	241	118** (49.0)	42.5 – 55.5	112** (46.5)	40.0 – 53.0	39 (16.2)	11.8 – 21.5	27** (11.2)	7.5 – 15.9
	Part–time	112	72 (64.3)	54.7 – 73.1	68 (60.7)	51.0 – 70.0	26 (23.2)	15.8 – 32.1	24 (21.4)	14.2 – 30.2
	Not-working	44	27 (61.4)	45.5 – 75.6	27 (61.4)	45.5 – 75.6	8 (18.2)	8.2 – 32.7	5 (11.4)	3.8 – 24.6
Wife’s occupation	Housewife	264	141 (53.4)	47.2 – 59.5	134 (50.8)	44.6 – 56.9	45 (17.0)	12.7 – 22.1	35 (13.3)	9.4 – 18.0
	Employed	136	77 (56.6)	47.9 – 65.1	74 (54.4)	45.7 – 63.0	28 (20.6)	14.1 – 28.4	21 (15.4)	9.8 – 22.6
Monthly wage	<10MR	100	61* (61.0)	50.7 – 70.6	55* (55.0)	44.7 – 65.0	21 (21.0)	13.5 – 30.3	21**(21.0)	13.5 – 30.3
	10–20MR	138	78 (56.5)	47.8 – 64.9	77 (55.8)	47.1 – 64.2	26 (18.8)	12.7 – 26.4	21 (15.2)	9.7 – 22.3
	20–50MR	102	54 (52.9)	42.8 – 62.9	52 (51.0)	40.9 – 61.0	20 (19.6)	12.4 – 28.6	12 (11.8)	6.2 – 19.6
	> 50MR	60	25 (41.7)	29.1 – 55.1	24 (40.0)	27.6 – 53.5	6 (10.0)	3.8 – 20.5	2 (3.3)	0.4 – 11.5
Argument in wife’s family	Never	152	64**(42.1)	34.2 – 50.4	60**(39.5)	31.6 – 47.7	14** (9.2)	5.1 – 15.0	15** (9.9)	5.6 – 15.8
	Sometimes	193	117 (60.6)	53.3 – 67.6	113 (58.5)	51.3 – 65.6	46 (23.8)	18.0 – 30.5	27 (14.0)	9.4 – 19.7
	Often	54	37 (68.5)	54.4 – 80.5	35 (64.8)	50.6 – 77.3	13 (24.1)	13.5 – 37.6	14 (25.9)	15.0 – 39.7
Argument in spouse’s family	Never	126	46**(36.5)	28.1 – 45.6	42**(33.3)	25.2 – 42.3	8** (6.3)	2.8 – 12.1	12** (9.5)	5.0 – 16.0
	Sometimes	218	127 (58.3)	54.4 – 64.9	124 (56.9)	50.0 – 63.4	45 (20.6)	15.5 – 26.6	30 (13.8)	9.5 – 19.1
	Often	55	45 (81.8)	69.1 – 91.0	42 (76.4)	63.0 – 86.8	20 (36.4)	23.8 – 50.4	14 (25.5)	14-39.0

* means P-value <0.25, ** means P-value <0.05 (significant variables are indicated by star signs in the first row). P–value <0.25 is assumed as the level of selection for logistic regression analysis. MR = Million Rial

Among all participants, 61 (15.3%) women reported being ignored or treated indifferently more than 3 times in the past 12 months, which was the highest frequency in mental violence, as well as amongst all the questions asked. The second most frequent mental violence experienced, was being insulted and felt bad about themselves more than 3 times within the past 12 months (57 (14.2%) women). The least answered question in the mental violence was given by 34 women (8.5%) about their husband often feeling suspicious of them being faithful during their lifetime.

Being pushed or shoved and also being slapped or had something thrown at more than 3 times in the last 12 months, was the most frequent answers among physical violence questions (22 (5.5%) and 19 (4.8%) women), respectively.

The least answered questions were being choked or burnt on purpose with a frequency of 6 (1.5%) during their lifetime.

A total of 21 (5.3%) women were forced to have intercourse without their consent more than 3 times in the last 12 months, and 25 (6.3%) had this experience 2-3 times in the past 12 months, which was the highest in sexual violence. The participants’ answers to the questions are shown in Appendix 2.

**Appendix 2:** Answers received from participants.

Answers Questions	Never	Yes but not within 12months	Once (recent 12months)	2–3 times (recent 12months)	More than three times (recent 12month)
Question 1	332 (83.0%)	7 (1.8%)	5 (1.3%)	26 (6.5%)	30 (7.5%)
Question 2	321 (80.3%)	10 (2.5%)	12 (3.0%)	19 (4.8%)	38 (9.5%)
Question 3	295 (73.8%)	11 (2.8%)	5 (1.3%)	28 (7.0%)	61 (15.3%)
Question 4	319 (79.8%)	11 (2.8%)	14 (3.5%)	26 (6.5%)	30 (7.5%)
Question 5	366 (91.5%)	5 (1.3%)	7 (1.8%)	6 (1.5%)	16 (4.0%)
Question 6	320 (80.0%)	12 (3.0%)	14 (3.5%)	20 (5.0%)	34 (8.5%)
Question 7	338 (84.5%)	3 (0.8%)	6 (1.5%)	24 (6.0%)	9 (7.2%)
Question 8	299 (74.8%)	5 (1.3%)	5 (1.3%)	34 (8.5%)	57 (14.2%)
Question 9	320 (80.0%)	4 (1%)	14 (3.5%)	25 (6.3%)	37 (9.3%)
Question 10	319 (79.8%)	9 (2.3%)	10 (2.5%)	27 (6.8%)	35 (8.8%)
Question 11	366 (91.5%)	4 (1.0%)	6 (1.5%)	10 (2.5%)	14 (3.5%)
Question 12	345 (86.3%)	9 (2.3%)	15 (3.8%)	12 (3.0%)	19 (4.8%)
Question 13	344 (86.0%)	9 (2.3%)	6 (1.5%)	19 (4.8%)	22 (5.5%)
Question 14	370 (92.5%)	5 (1.3%)	4 (1.0%)	5 (1.3%)	16 (4.0%)
Question 15	374 (93.5%)	4 (1.0%)	7 (1.8%)	4 (1.0%)	11 (2.8%)
Question 16	394 (98.5%)	0 (0.0%)	2 (0.5%)	1 (0.3%)	3 (0.8%)
Question 17	390 (97.5%)	0 (0.0%)	4 (1.0%)	1 (0.3%)	5 (1.3%)
Question 18	347 (86.8%)	2 (0.5%)	5 (1.3%)	25 (6.3%)	21 (5.3%)
Question 19	383 (95.8%)	1 (0.3%)	2 (0.5%)	7 (1.8%)	7 (1.8%)
Question 20	383 (95.8%)	2 (0.5%)	3 (0.8%)	8 (2.0%)	4 (1.0%)

1. Frequency and percentages of participants’ answers are considered in this table.

According to the multivariable analysis, women who were not living with their partner (divorced, separated) experienced overall DV 6.5 (95%CI: 2.1, 20.2) times, mental 5.6 (95%CI: 1.8, 16.9) times, and physical DV 5.2 (95%CI: 2.2, 12.4) times more than women who were living with their spouses. Wives, whose spouses were in their 30-49 or older than 50 years experienced mental violence 2.7 (95%CI: 1.3, 5.6), and 3.8 (95%CI: 1.7, 8.8) times more than those younger than 30 years. Women from families with 3 or more children reported to have experienced 3.8 (95%CI: 1.8, 7.9) and 4.6 (95%CI: 1.8, 11.9) times more DV in general and physical violence compared to those from families without children, respectively (Table 2).

**Table 2:** The results of the logistic regression analysis.

Type of violence	Status		Adjusted OR(95% CI up., low.)	Unadjusted OR(95% CI up., low.)
Total	Marital status	Living together	Reference	
		Living separated	6.4 ** (2.1–20.2)	4.8 ** (1.8–12.8)
		0	Reference	
	Number of children	1 child	2.3 ** (1.2–4.4)	1.8 * (1.0–3.2)
		2 children	2.7 ** (1.5–5.1)	1.7 (1.0–2.9)
		> 3 children	3.8 ** (1.8–7.9)	2.3 * (1.2–4.4)
	House ownership	Owning	Reference	
		Not owning	2.6 ** (1.6–4.0)	2.1 ** (1.4–3.1)
		Never	Reference	
	Argument in spouse’s family	Sometimes	2.2 ** (1.4–3.5)	2.4 ** (1.5–3.8)
		Often	8.5 ** (3.8–19.2)	7.8 ** (3.6–17.0)
Mental	Marital status	Living together	Reference	
		Living separated	5.6 ** (1.8–16.9)	5.3 ** (2.0–14.2)
		< 30	Reference	
	Spouse’s age	30 – 49	2.7 ** (1.3–5.6)	1.9 * (1.0–3.7)
		> 50	3.8 ** (1.7–8.8)	2.1* (1.0–4.4)
	House ownership	Owning	Reference	
		Not owning	2.7 ** (1.7–4.3)	2.1 ** (1.4–3.2)
	Argument in spouse’s family	Never	Reference	
		Sometimes	2.3 ** (1.4–3.7)	2.6 ** (1.7–4.2)
		Often	6.9 ** (3.2–14.8)	6.5 ** (3.1–13.3)
Physical	Marital status	Living together	Reference	
		Living separated	5.2 ** (2.2–12.4)	4.3 ** (2.0–9.3)
		0	Reference	
	Number of children	1 child	2.1 (0.9–5.2)	1.8 (0.8–4.1)
		2 children	2.8 * (1.2–6.8)	2.0 (0.9–4.6)
		> 3 children	4.6 ** (1.8–11.9)	3.5 ** (1.5–8.2)
		Never	Reference	
	Argument in wife’s family	Sometimes	2.3 * (1.1–5.2)	3.1 ** (1.6–5.9)
		Often	1.5 (0.6–4.0)	3.1 ** (1.4–7.2)
		Never	Reference	
	Argument in spouse’s family	Sometimes	2.2 (0.9–5.5)	3.8 ** (1.7–8.4)
		Often	6.6 ** (2.3–18.7)	8.4 ** (3.4–20.8)
Sexual	Monthly wage	<10 M	9.0 ** (2.0–40.8)	7.7 ** (1.7–34.2)
		10M – 20M	6.089 * (1.4–27.3)	5.2 * (1.2–23.0)
		20M – 50M	4.551 (1.0–21.5)	3.9 (0.8–17.9)
		>50M	Reference	
		Never	Reference	
	Argument in wife’s family	Sometimes	1.6 (0.8–3.1)	1.5 (0.8–2.9)
		Often	3.7 ** (1.6–8.6)	3.2** (1.4–7.2)

OR, odds ratio; Dash marks are insignificant factors in previous chi-square analysis. * means P–value <0.05 and ** means P–value <0.01. MR = Million Rials

## Discussion

In this study, in Shiraz, southwestern Iran, more than half of the women had experienced DV at least once in their lifetime. This was similar to the prevalence reported by a previous study in Shiraz and Rafsanjan.^8,15^ According to the findings from the WHO multi-country study conducted by Garcia-Moreno et al.

Bangladesh, Peru and the United Republic of Tanzania had similar prevalence, while Ethiopia had a total prevalence of 70%.^5,16^ However, a meta-analysis study conducted on 31 articles on domestic violence against women (from 2000 to 2014) estimated that the total prevalence of this phenomenon in Iran was 66% while DV prevalence in the southern regions of the country was reported to be 70% in the same study.^6^ These different fin dings could be due to different cul-tures and traditions by illustrating men superiority across different regions.^6,16^

The highest prevalence in this study was found in mental violence with 52%. Despite the differences in cultural, religious, economic and ethnic factors, the evi-dence is in favour of mental violence as the major do-mestic violence experienced all over the world.^7,17,18^ The number of individuals in the present study who only experienced sexual and/or physical violence without any mental harm was scarcely low. Nonetheless, there are some studies in favor of physical violence, having a greater burden on public health. A study by Coker et al. reported 77.3% physical or sexual and 22.7 % non-physical abuse.^19^ Moreover, in a study by Bonomi et al., depression rates from physical and sexual abuse were higher than non-physical abuse compared to never abused women.^20^ Accordingly, more studies should be conducted to clarify the most detrimental type of violence to implement preventive programmes and reduce its possible negative impacts.

WHO multi-country survey also showed that from all injured women, 86% had experienced at least one se-vere physical violence, and only 14% experienced moderate violence. Whereas, compared to our study, moderate and severe violence in injured women was 49% and 51%, respectively. This shows that women suffering from domestic violence experienced moderate and severe physical violence equally. This could be due to cultural and traditional gender norms, which supports beating up women in some regions.^5,16^

Additionally, it was concluded that the majority of women were ignored or treated indifferently by their husbands. Similarly, a study in Esfahan, Iran reported this action as the highest violence experienced by women.^21^ Our finding concludes that women in Shiraz are mostly harmed by being ignored by their husbands, and this characteristic might be acquired during life through the family and society. Thus, public health organizations should implement strategies to improve family communi-cation and relationship via public awareness educational programs at workplaces as well as the society namely, public transport advertisements and billboards.^19,20,22,23^

According to this study, living separately significantly increased the risk of domestic violence (above 5 times). Similarly, a study conducted in Sweden indicated that pregnant women living separately were more likely to experience domestic violence.^24^ Nonetheless, a study in Portugal, revealed intimate partner violence in dating couples compared to married couples, and reported general disapproval of violence as well as increased support among the married partici-pants. ^25^

As it was stated, spouse age was related to mental violence in this research. Similarly, a study in Egypt re-vealed more violence in men over 40 years.^26^ However, studies by Izmirli 2014 in Turkey and Adebowale 2018 in Nigeria showed that violence was higher among younger men.^27,28^

In line with most previous studies, it was revealed that women in families with more children were more likely to experience physical DV.^29-31^ Whereas, a study by Ahmadi et al. (2016) revealed that more partner violence was experienced by women in families with no children. ^17^ This could be due to increase in family management problems and challenges for a satisfying life, which can contribute to spouse confusion and anger, leading to violence against their wives. Consequently, educational strategies should be considered by organizations to aid families tackle stressful challenges, by prioritizing their needs.^22,23^

Total and mental DV against women approximately doubled in families who lived in rented houses compared to families who owned a house. Moreover, those with monthly low and low-middle income compared to high-income families experienced sexual violence more than 9 and 6 times, respectively. Previous studies also reported increasing significant effects of different socio-economic related variables on DV. ^16,17^ According to a survey conducted in sub-Saharan Africa, women living in rich families in Zambia and Mozam-bique experienced more partner violence whereas, in Zimbabwe and Kenya, it was higher among women coming from poor households. In the same study, women from the middle class in Nigeria and Cameroun experienced more partner violence compared to poor and rich families. ^32^ This is in contrast with a survey conducted in Eastern India, reporting less DV in families with higher income.^33^ Accordingly, socio-economic or better said, family income is a correlate of DV, but the direction of its effect seems to be cultural dependent. However, financial issues should be addressed by responsible organizations by providing more affordable housing to families as well as supplemental food assistance programs. In addition, families should be well educated to manage their routine financial matters more efficiently^34,35^

One of the most effective factors worth mentioning is the husbands who had often witnessed their parent’s arguments. It can be said that DV was approximately 6 to 8 times more prevalent in these families. In the same manner, women who experienced or witnessed DV in their parents’ house were more susceptible to this phenomenon during their marital life. Our study is in line with a study by Holt et al., 2008 revealing an increase in behavioural and emotional problems of children who had witnessed DV throughout their lives.^36^ The study conducted by Krug et al. reported that children witnessing DV were more likely to develop various mental problems and engage in interpersonal violence as they grow older. It was also reported that childhood exposure to violence is a risk factor for many behavioural disorders.^37^ Also, surveys by Locascio M. in 2018 and Yount et al., 2006 revealed a positive relationship between women being victims of childhood psychosocial abuse and domestic violence.^31,38^ It can be suggested that by monitoring youth mental health regularly during their education, early approach could be implemented in order to reduce the negative effects of family arguments on their behavioral development. Moreover, incorporating wellness activities in the school curriculum will certainly have beneficial impacts on this issue.^19,20,23^

Also, a study in Jahrom, Iran showed that younger women and marital years less than 5 years tend to experience DV more, which might be due to their disorientation on how to cope with family problems and con-front their husbands’ violent behaviour. Ahmadi et al. also concluded that younger women experience more physical violence than older women.^17^ Similarly, a study in 34 countries, 2017 showed in-creased DV among younger women.^39^

**Limitations**

Due to the sensitivity of some questions, the time spent on each question was approximately 10 minutes, but collecting data on some confounding variables was not possible. Nevertheless, efforts were made to obtain data for important variables.

In terms of sampling, a door-to-door survey was not feasible since many families live with their husband’s parents, especially in low socio-economic areas. However, although public and private clinics in all municipality districts were considered in this study, women visiting healthcare centres might not completely be a representative of the total population.

Additionally, due to recall and prestige bias, some answers might not be fully truthful. However, efforts were made to maintain confidentiality by providing private rooms for answering the questions as well as closed boxes for placing questionnaires. Moreover, the necessity of these studies in reducing the prevalence of DV was explained with details to participants, and many were interestingly grateful for being asked about their situation.

## Conclusion

According to multiple research, public mental health has a significant dependency on people's behaviours and their roles in the environment, which can mainly be achieved through family mental health.^5,37^ As WHO reports, early childhood interventions and family therapy can reduce the long-term effects of DV on children, with a significant effect on their future lives.^36^ Also, economic security can significantly affect men's behaviour towards their wives, which should be considered.^40^ Ultimately, considering these essential elements and implementing preventive strategies for all family member are highly necessary in this region.

As it was mentioned above, DV has a significant role in reducing each family member’s personal capabilities, since it can lead to depression, anxiety, physical and mental abnormalities, and suicidal thoughts.^8^ The outcomes can contribute to a poor social and public performance of family members, leading to an insecure and harmful society.^36^ Thus, this research was developed, and different variables were examined to help improve family and public health by focusing on finding out the related elements. Finally, it can be said that such studies play a crucial role in developing public health strategies with aim to improve family relationships and child development. 

**Acknowledgements**

We gratefully acknowledge the 400 participants who shared their experiences with us, which can surely help society’s health to a great extent. Also, we would like to thank all the staff and healthcare workers who dedicated their time to the present study.

## References

[1] Taherkhani S, Mirmohammadali M, Kazemnejad A, Arbabi M (2010). Association experience time and fear of domestic violence with the occurrence of depression in women. Iranian Journal of Forensic Medicine.

[2] Nojomi M, Agaee S, Eslami S (2007). Domestic violence against women attending gynecologic outpatient clinics. Archives of Iranian Medicine.

[3] Othman S, Mat Adenan NA (2008). Domestic violence management in Malaysia: A survey on the primary health care providers. Asia Pac Fam Med.

[4] Rezaei A, Khodadadi Z, Mirmohamadi L (2011). On the Relationship between abused spouses’ dysfunctional thoughts and the tendency towards suicidal thoughts. Scientific Journal Management System.

[5] Garcia-Moreno C1, Jansen HA, Ellsberg M, Heise L, Watts CH; WHO Multi-country Study on Women's Health and Domestic Violence against Women Study Team. Prevalence of intimate partner violence: findings from the WHO multi-country study on women's health and domestic violence. Lancet. 2006;368(9543):1260-9. 10.1016/S0140-6736(06)69523-8 17027732

[6] Hajnasiri H, Ghanei Gheshlagh R, Sayehmiri K, Moafi F, Farajzadeh M (2016). Domestic Violence Among Iranian Women: A Systematic Review and Meta-Analysis. Iran Red Crescent Med J.

[7] Kargar Jahromi M, Jamali S, Rahmanian Koshkaki A, Javadpour S (2016). Prevalence and Risk Factors of Domestic Violence Against Women by Their Husbands in Iran. Glob J Health Sci.

[8] Shayan A, Masoumi SZ, Kaviani M (2015). The Relationship between Wife Abuse and Mental Health in Women Experiencing Domestic Violence referred to the Forensic Medical Center of Shiraz. Journal of Education and Community Health.

[9] Akhondzadeh S (2019). Possibility for science without borders in the Trump era. The Lancet.

[10] Habibzadeh F (2018). Economic sanction: a weapon of mass destruction. Lancet.

[11] Iran SCo. 2016 National Population and Housing Census. 2016, https://www.amar.org.ir, accessed 10 May 2019.

[12] García-Moreno C, Jansen H, Ellsberg M, Heise L, Watts C (2005). WHO multi-country study on women’s health and domestic violence against women. Geneva: World Health Organization.

[13] Nouhjah S, Latifi SM (2014). Variation in the Prevalence of Domestic Violence between Neighboring Areas. International Scholarly Research Notices.

[14] Molavi Vardajani H, Haghdoost AA, Shahravan A, Rad M (2016). Cleansing and preparation of data for statistical analysis: A step necessary in oral health sciences research. J Oral Health Oral Epidemiol.

[15] Torkashwand F, Rezaeean M, Sheikhfathollahi M, Mehrabian M, Bidaki R, Garousi B (2013). The Prevalence of the Types of Domestic Violence on Women Referred to Health Care Centers in Rafsanjan in 2012. Journal of Rafsanjan University of Medical Sciences.

[16] Semahegn A, Belachew T, Abdulahi M (2013). Domestic violence and its predictors among married women in reproductive age in Fagitalekoma Woreda, Awi zone, Amhara regional state, North Western Ethiopia. Reproductive Health.

[17] Ahmadi R, Soleimani R, Jalali MM, Yousefnezhad A, Roshandel Rad M, Eskandari A (2017). Association of intimate partner violence with sociodemographic factors in married women: a population-based study in Iran. Psychol Health Med.

[18] Houry D, Kemball R, Rhodes KV, Kaslow NJ (2006). Intimate partner violence and mental health symptoms in African American female ED patients. Am J Emerg Med.

[19] Coker AL, Smith PH, McKeown RE, King MJ (2000). Frequency and correlates of intimate partner violence by type: physical, sexual, and psychological battering. Am J Public Health.

[20] Bonomi AE, Thompson RS, Anderson M, Reid RJ, Carrell D, Dimer JA (2006). Intimate partner violence and women's physical, mental, and social functioning. Am J Prev Med.

[21] Mousavi SM, Eshagian A (2005). Wife abuse in Esfahan, Islamic Republic of Iran, 2002. East Mediterr Health J.

[2] Campbell JC, Manganello J (2006). Changing Public Attitudes as a Prevention Strategy to Reduce Intimate Partner Violence. Journal of Aggression, Maltreatment & Trauma.

[23] Coker AL (2004). Primary prevention of intimate partner violence for women's health: a response to Plichta. J Interpers Violence.

[24] Finnbogadottir H, Dykes AK (2016). Increasing prevalence and incidence of domestic violence during the pregnancy and one and a half year postpartum, as well as risk factors: -a longitudinal cohort study in Southern Sweden. BMC Pregnancy and Childbirth.

[25] Machado C, Martins C, Caridade S (2014). Violence in Intimate Relationships: A Comparison between Married and Dating Couple. J Journal of Criminology.

[26] Ali R, Radwan R (2017). Magnitude and determinants of domestic violence against ever married women in Sohag, Egypt. International Journal of Medical Science and Public Health.

[27] Izmirli GO, Sonmez Y, Sezik M (2014). Prediction of domestic violence against married women in southwestern Turkey. Int J Gynaecol Obstet.

[28] Adebowale AS (2018). Spousal age difference and associated predictors of intimate partner violence in Nigeria. BMC Public Health.

[29] Flake DF (2005). Individual, family, and community risk markers for domestic violence in Peru. Violence Against Women.

[30] Graham K, Bernards S, Laslett AM, Gmel G, Kuntsche S, Wilsnack S, et al. Children, Parental Alcohol Consumption, and Intimate Partner Violence: A Multicountry Analysis by Perpetration Versus Victimization and Sex. J Interpers Violence. 2018 Oct 17:886260518804182. 10.1177/0886260518804182 PMC6470056 30328365

[31] Yount KM, Carrera JS (2006). Domestic Violence against Married Women in Cambodia. Social Forces.

[32] Bamiwuye SO, Odimegwu C (2014). Spousal violence in sub-Saharan Africa: does household poverty-wealth matter?. Reprod Health.

[33] Babu BV, Kar SK (2010). Domestic violence in Eastern India: factors associated with victimization and perpetration. Public Health.

[34] Chilton MM, Rabinowich JR, Woolf NH (2014). Very low food security in the USA is linked with exposure to violence. Public Health Nutr.

[35] Kim JC, Watts CH, Hargreaves JR, Ndhlovu LX, Phetla G, Morison LA (2007). Understanding the impact of a microfinance-based intervention on women's empowerment and the reduction of intimate partner violence in South Africa. Am J Public Health.

[36] Holt S, Buckley H, Whelan S (2008). The impact of exposure to domestic violence on children and young people: a review of the literature. Child Abuse Negl.

[37] Krug EG, Mercy JA, Dahlberg LL, Zwi AB (2002). The world report on violence and health. Lancet.

[38] LoCascio M, Infurna MR, Guarnaccia C, Mancuso L, Bifulco A, Giannone F. Does Childhood Psychological Abuse Contribute to Intimate Partner Violence Victimization? An Investigation Using the Childhood Experience of Care and Abuse Interview. Journal of Interpersonal Violence. 2018:886260518794512. 10.1177/0886260518794512 30136884

[39] Kidman R (2017). Child marriage and intimate partner violence: a comparative study of 34 countries. International Journal of Epidemiology.

[40] Cunradi CB, Caetano R, Schafer J (2002). Socioeconomic Predictors of Intimate Partner Violence Among White, Black, and Hispanic Couples in the United States. Journal of Family Violence.

